# Usability of Particles Made from Lesser-Used European Wood Species Mixed with Spruce Particles in the Particleboard Core Layer

**DOI:** 10.3390/polym17101291

**Published:** 2025-05-08

**Authors:** Roman Reh, Jan Izdinsky, Dominik Hrusovsky, Pavel Kral, Tomas Pipiska, Miroslav Jopek

**Affiliations:** 1 Department of Wood Technology, Faculty of Wood Sciences and Technology, Technical University in Zvolen, Masaryka 24, 96001 Zvolen, Slovakia; jan.izdinsky@tuzvo.sk (J.I.); dominik.hrusovsky10@gmail.com (D.H.); 2 Faculty of Forestry and Wood Technology, Mendel University in Brno, Zemedelska 1665/1, 613 00 Brno, Czech Republic; kral@mendelu.cz (P.K.); tpipiska@gmail.com (T.P.); 3 Faculty of Mechanical Engineering, Brno University of Technology, Technicka 2, 616 69 Brno, Czech Republic; jopek@fme.vutbr.cz

**Keywords:** particleboard, raw materials, alder, birch, larch, physical properties, mechanical properties, density profiles

## Abstract

The effects of produced wood particles from three wood species—alder, birch, and larch—added in various amounts in the particle mixture consisting of spruce particles and three tested wood species in a particleboard core layer on selected physical and mechanical properties of particleboard were studied. In a laboratory, 16 mm thick three-layer urea–formaldehyde (UF)-bonded particleboards were produced at 5.23 MPa, 240 °C, and with a 10 s/mm pressing factor. Two particleboard surface layers consisted of fine spruce particles. In the particleboard core layer, spruce particles were combined with particles from alder, birch, and larch. The tested particleboards containing alder, birch, and larch were characterized by approximately identical thickness swelling and they met the requirements of the conventional values stated by major particleboard manufacturers of 8–10%. The tested particleboard in all variations met the minimum strength value P2 particleboard in three-point bending, which is conventionally set at 11.0 MPa. The tested particleboard also exceeded the required values of modulus of elasticity in bending and internal bond strength. Analysis of the relationships demonstrated by the density profile confirmed that all three investigated wood species are usable in a mixture of core particles of high-quality particleboard in the recommended amounts (10, 15, 20%).

## 1. Introduction

There is a long-term tendency to use spruce for particleboard production [1,2]. The European Panel Federation EPF states in the introduction to its web page that “Wood particles comprise the bulk of particleboard and are prepared in a mechanical chipper generally from coniferous softwoods, principally spruce” [3].

Aksenov et al. [4] presented a detailed analysis of the global particleboard production market. They compare production dynamics in countries with different levels of economic development and examine the contribution of major particleboard producers, such as China, the United States, Russia, and European Union countries, to global production. They concentrate on the factors driving the increased demand for particleboard and they also discuss future development prospects for the industry, including possible scenarios of growth or stagnation in production depending on global economic conditions, changes in raw material prices, and product demand. Their detailed study shows that the production volumes and consumption of particleboard will grow in the coming years definitely. And, therefore, more and more wood raw material will be needed for their production. Spruce will therefore be in demand, especially in areas of the temperate climate zone, but not only there.

The continuous development of particleboard production and the gradually deepening knowledge of the connections between their properties, raw material, and technological factors have pointed out the importance of the wood quality and the wood particles produced from wood, their morphology, geometric dimensions, and slenderness ratio. This fact, together with the current level of machinery for the production of wood particles, has led to the conclusion and recommendations of which wood species are the most suitable raw material for the production of particleboard [5,6].

Softwoods (coniferous) are more suitable for particleboard, primarily due to their low density, and they are in a range from which high-quality particles can currently be produced. The resulting demands and requirements for the raw material used are directed towards wood ranges from which definable, relatively highest-quality particles can be produced in a single-stage process [7,8].

In connection with the required low density of particleboard, mainly light coniferous woods are traditionally used for their production. However, the gradually expanding particleboard production began to feel the shortage of light coniferous raw materials; therefore, considerations began to be made about processing hard coniferous and soft and hard deciduous woods, despite their certain disadvantages and specific properties [5,9]. The uniform quality of particleboard produced from woods of different properties (density, structure, pH value, etc.) is required, and constant mixing ratios are assumed not only of individual woods but also of assortments for which specific technological conditions (adhesive and its amount, hydrophobization, pressing technique, particleboard density, etc.) are determined with regard to the required particleboard properties [10].

From the point of view of the particleboard quality, spruce is an extremely suitable wood for the particleboard production due to its properties [11]. Spruce belongs to the softer and lighter types of wood species, its density is around 350–400 kg/m^3^, but at the same time, it is strong, flexible, and it splits well [11,12]. Its surface is smooth, it is easy to work, and mainly it especially disintegrates well. Spruce wood is characterized by a creamy white color which can turn into a light brown, and it has no heartwood color. Therefore, its color also makes it suitable for the production of particleboard. Its advantage is that it is simple and fast drying [13,14,15].

Research, scientific, and technological workers in the particleboard industry ask themselves the following question: can spruce wood be replaced in particleboard in such a way that the particleboard quality is maintained, and production costs are not increased? Research and experiments are carried out (sometimes unfortunately by the system of trial and error) and, in addition to reasonable solutions, there is also the processing of unsuitable raw materials for particleboard production, which will cause more harm than good [10,16,17].

Currently, there are three basic sources of wood raw materials for particleboard production: (a) forest assortments, (b) industrial waste or residues from industrial wood processing, and (c) old wood, that is, wood from old, discarded wood products labeled as used wood or recycled wood, always appropriately sorted and treated [10,18,19]. In this research, we focused on the use of forest assortments, but from lesser-known and less-used European wood species for particleboard production.

Undeniably, these are important questions and solutions, because particleboards are a crucial product of the major wood-based products in global trade. Interest and consumption of particleboard are growing, well deservedly, as it is produced all over the world. It is a modern engineered wood product manufactured from wood particles mostly bonded with synthetic resin under exposure to a hot press at a certain pressure and temperature, with adequate properties especially for furniture production. In large volumes on modern high-capacity lines, it is possible to produce particleboard at reasonable prices, so we are talking about suitable panel materials with the perspective of production growth [1,9,13,14,16].

And besides, the particleboard production volume is already high even in the present; according to FAO statistics, the global market for particleboard reached a total value of more than 116,000,000 m^3^ in 2023, the European market for particleboard reached a total value of almost 44,000,000 m^3^ in 2023 according to FAO statistics, and it is mainly based on the processing of spruce definitively [20]. According to the IMARC Group, the global particleboard market size reached USD 23.8 billion in 2024 [21]. Looking forward, the IMARC Group expects the market to reach USD 31.7 billion by 2033, exhibiting a growth rate of 3.2% during 2025–2033 [21]. Upcoming construction and infrastructure projects are projected to generate more opportunities in the future for the European particleboard market [22].

It is therefore meaningful to deal with the high-quality replacement of a certain share of the spruce raw material in particleboard, and this was the purpose of this research. We wanted to go the route of exclusively replacing a certain percentage of the spruce raw material with wood raw material, namely (a) to propose for use only those alternative wood species that are found in forest reserves in interesting quantities [23,24,25,26,27,28], because conducting research with uninteresting reserves of some wood species in forests for particleboard or specifically with wood species with an exceptional minority occurrence in parks, orchards, or gardens is not interesting for economic practice [29,30,31]; (b) to use certain wood assortments in the particleboard core layer only [32,33]; this is so that both particleboard surfaces remain composed only of spruce and do not resemble any alternative raw materials in appearance and so that they do not cause any technological problems with the assumed dominant surface treatment of most particleboards produced for interior purposes with decorative foils.

We asked ourselves some time ago the question regarding which lesser known and less-used wood species would be suitable for their partial application in the particleboard core layer, and we attempted to answer it in our paper “Perspectives on Using Alder, Larch, and Birch Wood Species to Maintain the Increasing Particleboard Production Flow” [34]. Through our literature search activities, we sought to select such wood species that would be an example, through their properties, of the possible processing of other similar wood species for the identical purpose of replacing some spruce particles in the particleboard core layer. Through a detailed analysis of the characteristics and assessment of sufficient stock in the forests, the following wood species were selected: larch (*Larix decidua* Mill.) as a representative of possible softwood (coniferous) species, birch (*Betula pubescens* Ehrh.) as a representative of possible hardwood (deciduous) species with higher density, and alder (*Alnus incana* (L.) Moench) as a representative of possible hardwood (deciduous) species with lower density [24,25,26,27,28,35].

In the more forested countries of Central Europe (Slovakia, Czechia, Germany, Austria, Poland, etc.), alder is present in forest stands at a level of 2 to 3% of the total forest area. Data on the processed volumes of alder wood (but also birch and larch wood) vary depending on the annual production, demand, and forestry policy of individual countries, but it seems that in addition to various methods of its processing and use (production of veneer, plywood, furniture, cladding, smaller wood products, and decorative elements), an interesting amount of alder wood remains unused, burned, or not processed in the forests. Birch is present in forest stands in Central European countries in similar percentages (2–3.5%), with the exception of Poland, where the forest stands are represented to a greater extent (around 7%), which is related to the more northern location of this country and the gradual transition of coherent forests to the Baltic countries, where extensive birch stands are present and they are used mainly for the production of veneers and plywood. In this case too, an interesting amount of birch wood remains unused in the forests, is not processed, or is burned. Larch is also present in the forests in the monitored Central European countries, specifically at the level of 3–5% of the total forest area. Better larch assortments have a strong tradition of use in construction due to their durability and resistance to weathering; other assortments have less use and there exists their potential for the production of particleboard as well [23,24,25,26,27,28,35].

Based on detailed research of the physical, mechanical, and other properties of the native wood of the three selected wood species and on the basis of the previous experience with their processing cited in the literature, it appears that all three selected wood species are suitable for use in the particleboard core layer [34]. The effects of the produced core wood particles from the three wood species—alder, birch, and larch—added in various amounts to the particle mixture consisting of spruce particles, and three tested wood species on selected physical and mechanical properties of particleboard were studied for the paper.

## 2. Materials and Methods

### 2.1. Materials

#### 2.1.1. Wood Particles

Industrial spruce wood particles were bought directly from the factory of the company Kronospan Zvolen, Slovakia. Spruce particles were purchased in two qualities: (a) surface particles which we used directly for the purpose of the laboratory production of the two surface layers of particleboard without our intervention; the dimensions of the particles bought and used in the particleboard surface layers were from 0.125 to 1.0 mm, and (b) core particles which we used in the particle mix in our experiment (with alder, birch, and larch particles) for the laboratory production of the particleboard core layer. Spruce particles were purchased in a dried state, directly applicable for laboratory production (moisture content 1.75–2.50%).

Wood core particles from alder, birch, and larch were prepared in two production stages: (a) fresh alder, birch, and larch logs were purchased from the manufacturer, and they were wet chipped into a coarse chip fraction, using the grinding mill 230H Drum Mower (Klöckner KG, Hirtscheid-Erbach, Westerwaldkreis, Germany); and (b) subsequently, these coarse chips were transported to the production plant Kronospan Zvolen, Slovakia, in which core particles with the length of 0.25–4.0 mm were made from coarse chips. The chipping equipment Knife Ring Flakers G24 (Goos Engineering, s.r.o., Brno, Czech Republic) was used in Kronospan Zvolen, Slovakia, which is used in normal production, and it is regularly serviced and their knives are properly sharpened. Subsequently, all particles prepared for particleboard laboratory production were dried to a moisture content of 2% in the case of the core layer and to a moisture content of 4% in the case of the surface layers. We used a manual sorting device to sort particles according to size; the oversized fraction was sorted and excluded from the experiment. Fractions falling through a sieve with a mesh diameter of 0.125 mm were considered to be dust and it was excluded from the experiment as well. The sorting caused the particles used in the particleboard core layer to be from 0.25 to 4.0 mm in size.

#### 2.1.2. Adhesives

The urea–formaldehyde (UF) adhesive KRONORES CB 4005 D was used in the particleboard surface layers, and the UF adhesive KRONORES CB 1637 D was used in the particleboard core layer (Table 1). The UF adhesive was added to particles for the particleboard surface layers in an amount of 11%, and to particles for the particleboard core layer in an amount of 7%. Ammonium nitrate as a 55% water solution (2 or 4% of the dry mass of the UF adhesive in the case of the surface particles or core particles) was used as a hardener for the UF adhesive. A paraffin emulsion, with 37% dry mass, was applied on the surface and core particles in amounts of 0.6 and 0.7%, respectively.

### 2.2. Particleboard Preparation

**Figure 1:**
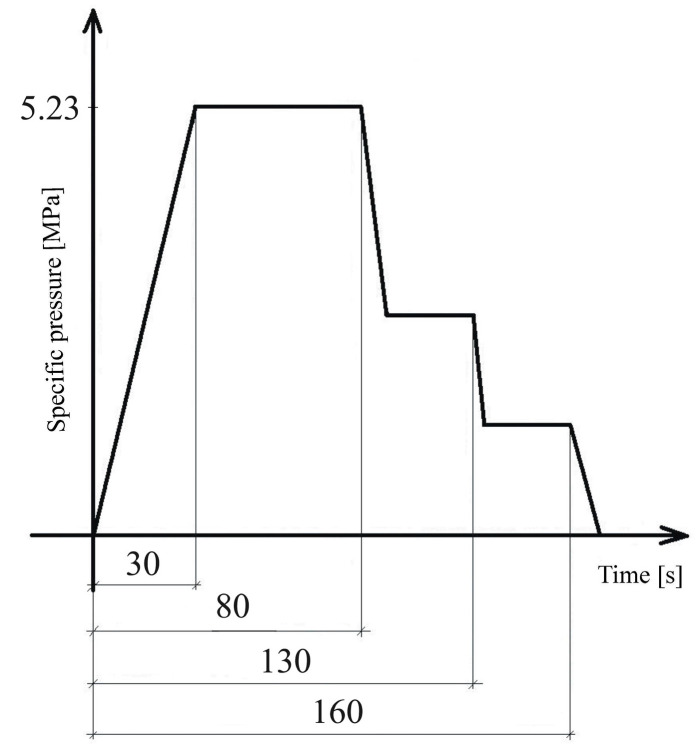
Standard three-stage pressing diagram for the manufacturing of particleboard.

Three-layer particleboards were produced under laboratory conditions. Particleboards were made with the dimensions of 400 × 300 × 16 mm, and their calculated, density condition at 20 °C and 65% relative humidity was 620 ± 10 kg/m^3^. UF adhesive with the indispensable hardener and paraffin emulsion were applied on the conditioned wood particles in a laboratory rotary mixing device (Technical University in Zvolen, Slovakia). The adhesive was applied by using a pressure of approximately 4.5 bar onto the falling stream of particles at the center of the rotating drum device. The moisture content of the wood particles for the surface layers after applying the adhesive was 9.7 to 10.5%, and the moisture content of the wood particles for the core layer after applying the adhesive was 6.1 to 6.9%. The particle mat was layered manually in relevant rectangular forms positioned on an aluminum siliconized plate to prevent adherence between the particleboard produced and the plate. The surface/core particle ratio was 33/67. The particle mat was (a) cold pre-pressed in a room-temperature environment at a pressure of 1 MPa before the rectangular form was removed; after removing the form, the particle mat was covered with a second aluminum siliconized plate and transferred to the laboratory hot press, and (b) pressed in the laboratory single-opening press CBJ 100-11 (TOS, Rakovník, Czech Republic); pressing was conducted in accordance with the pressing diagram (Figure 1) at a maximum temperature of 240 °C, a maximum pressure of 5.23 MPa. The pressing factor used for particleboard pressing was 10 s/mm. After hot pressing, particleboards were cooled at ambient conditions, then edge-trimmed, and finally stored in a climate chamber at 20 °C and 65% of relative humidity within 14 days before sample cutting. In total, 60 particleboards were manufactured, i.e., six of each type (Table 2).

### 2.3. Physical and Mechanical Properties of Particleboard

**Figure 2:**
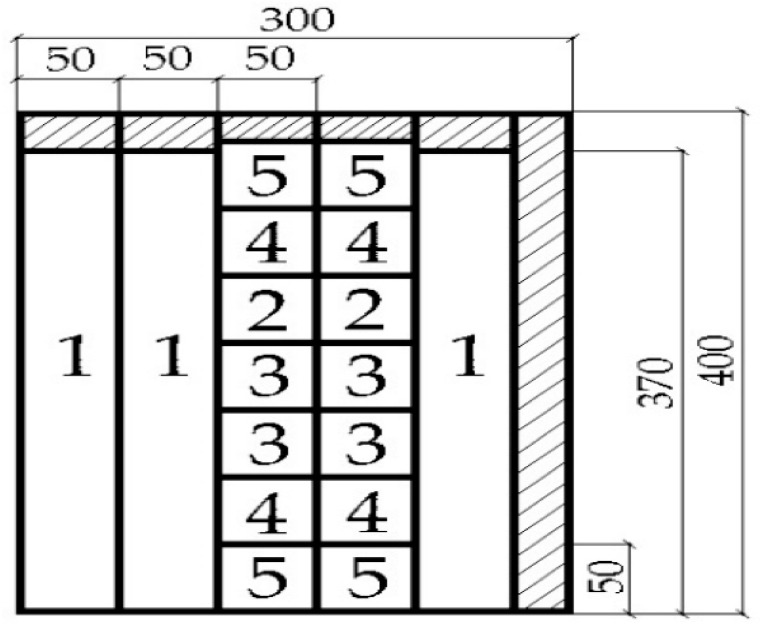
Cutting scheme for laboratory-manufactured particleboard: 1—samples for testing modulus of rupture and modulus of elasticity by 3-point bending test [43], 2—samples for testing thickness swelling and water absorption after 2 and 24 h [41,42], 3—samples for testing internal bond strength [44] and density [39], 4—samples for testing density profiles [46], and 5—spare samples.

The universal testing machine TiraTest 2200 (VEB TIW Rauenstein, Schalkau, Germany) was used to analyze the mechanical properties of the particleboard produced. The selected properties of the particleboard were determined according to the European standards (EN); in one case (since there is no EN), it was the Slovak technical standard (STN): the density by EN 323 [39], the moisture content by EN 322 [40], the thickness swelling and water absorption after 2 and 24 h by EN 317 [41] and STN 49 0164 [42], the modulus of rupture in bending and the modulus of elasticity in bending by EN 310 [43], and the internal bond strength (the tensile strength perpendicular to the plane) of the particleboard by EN 319 [44]. The samples for these tests were prepared from the 4-week air-conditioned particleboard (Figure 2). The classification of the particleboard was performed in accordance with the European standard EN 312 [45], considering the requirements of the particleboard (type P2), with a thickness ranging between 13 and 20 mm.

### 2.4. Density Profiles

Density profiles were measured on three 50 mm × 50 mm × 16 mm specimens cut from each panel (six panels for each wood species tested). Density profiles were obtained at an interval of 0.01 mm through the sample thickness using an X-ray density profile analyzer (DPX300-LTE, Imal, San Damaso, Italy), and on the basis of all measurements, an average curve (the average density profile) was calculated for each wood species of the examined particleboard.

### 2.5. Statistical Analyses

The statistical software STATISTICA 12 was used to analyze the gathered data. The descriptive statistics deal with the basic statistical characteristics of the studied properties—the arithmetic means and standard deviation. Simple linear correlation analysis together with the coefficient of determination was used as a method of inductive statistics to evaluate the measured data.

## 3. Results and Discussion

### 3.1. Physical Properties of Particleboard

The basic physical properties of particleboard are presented in Table 3, Table 4 and Table 5.

The individual proportions of wood and other components of the particleboard before pressing and subsequently the entire board-forming process in the laboratory were correct. The limit deviation of density from the average value of ±10%, according to EN 312 [45], is observed by numerous manufacturers, and we have not attacked it by far.

To compare densities, some factories do not like to publish the particleboard densities they produce (the companies state “Plant specific”), but depending on the type of particles used (in our case, spruce is the majority) and the board thicknesses, the density is usually approximately in the mentioned range [47,48]. Other factories publish density data in their datasheets and their data (depending on the type of particles used and board thickness) are consistent with the densities we produce [49,50,51,52]. That was the point: to produce such densities of the laboratory experimental boards that match the densities of commonly industrially produced particleboards.

All reached particleboard densities were in a very narrow interval. Their densities were not affected by the type of particles and amount apparently (Table 3, Table 4 and Table 5). This finding was also confirmed by the standard deviation values being lower than 20 as well as by the r^2^ values of the linear correlations being lower than 0.005 in all cases. This means that the tested moisture, physical, and mechanical properties of the particleboard could not be and were not affected by density.

The thickness swelling of particleboard after 2 h complies with the accustomed maximum value of 8%, and some factories set higher permitted values internally (10%) [51,52]. It is clear from Table 3, Table 4 and Table 5 that the swelling of the boards after 2 h is reliably below this limit for all monitored particle ratios. This feature is always influenced by the amount of hydrophobizing agent added in the particleboard production process and the fractional composition of the surface and core particles in particleboard; in any case, it is clear that the thickness swelling of the particleboard after 2 h is reliably satisfactory in all monitored cases (the values of all boards were only around 3–4%).

The thickness swelling of the particleboard using three less-used European wood species in their core layer after 24 h corresponds to the swelling of particleboard using exclusively spruce wood in the core layer. It seems as if higher proportions of birch in the core layer improve swelling after 24 h, which is probably due to the internal structure of birch as a hardwood with a higher density, and that in combination with the used adhesive, it did not swell as clearly as a lower density wood species. The positive effect of birch in various sheet composite products has also been recognized by other authors [9,53,54,55]. In any case, it is clear that the swelling of particleboard after 24 h is reliably satisfactory in all cases monitored.

The effect of the particles from three lesser-European wood species on the thickness swelling (after 2 and 24 h) of particleboard cannot be considered as crucial.

Water absorption of commonly produced particleboards is usually obvious, which is given by their internal structure. Due to the achieved water absorption values, particleboard products are a material suitable mostly for interiors with a dry environment only. This trend is also confirmed by the particleboard produced by us using three lesser-used European wood species in their core layer. Their water absorption values after 2 and 24 h are completely at the level of particleboard made from pure spruce particles, and therefore they can be reliably used in interiors under the same conditions as conventional particleboard.

Water absorption of particleboard is a somewhat underestimated property; sometimes it is not even mentioned in scientific and research papers on the physical properties of particleboard, and we do not have a uniform European standard with a uniform methodology for its research. We used the proven methodology of our older national standard. This methodology is focused on determining the amount of water the particleboard can absorb within a given time duration (after 2 and 24 h). The initial weight of samples and their final weight are determined, and the water absorption in “%” is calculated using the same formula as for the thickness swelling. Other authors use general formulas to determine which are capable of determining the amount of water that the particleboard can absorb within a given time duration. These are not unreliable tests, but they are acceptable [56,57,58,59].

The effect of the particles from three lesser-European wood species on the water absorption (after 2 and 24 h) of particleboard cannot be considered as significant as the r^2^ value of the linear correlations ranged only between 0.001 and 0.05.

### 3.2. Mechanical Properties of Particleboard

The basic mechanical properties of particleboard are presented in Table 6, Table 7 and Table 8.

The mechanical properties of the particleboard based on three wood species in their core layer in bending, i.e., the modulus of rupture and the modulus of elasticity, fulfilled the requirements for particleboard type P2 (according to EN 312 [45]), and they were not negatively influenced by three lesser-used European wood species (Table 6, Table 7 and Table 8). This finding was confirmed by the r^2^ values of the linear correlations being lower than 0.01 as well.

The minimum strength value of the three-layer P2 particleboard in three-point bending (modulus of rupture), according to the EN 312 standard, is set at 11.0 MPa. For all proportions of the examined wood species in the core layer, the minimum value of bending strength prescribed by the standard is met, similarly to that for the pure spruce particleboard. Table 7 shows that the addition of birch in the core layer has a positive effect on the bending strength of the entire particleboard, and boards with the addition of birch in the core layer show better bending strength than unmodified spruce particleboard at all three concentrations. This is probably due to the internal structure of birch as a hardwood with a higher density which, in combination with the used adhesive, created a stronger and more compact board material during the board-forming process by pressing. Even in this case, it is true that the positive effect of birch in various sheet composite products has also been recognized by other authors [9,53,54,55].

The minimum value of the modulus of elasticity for the P2 particleboard is set at 1600 MPa by EN 312. This minimum value was exceeded for all proportions of the wood species investigated in the particleboard core layer, which was already expected from the board results of the three-point bending strength. When comparing the individual proportions of the three investigated wood species with unmodified particleboard, the differences in the modulus of rupture are minimal (around 2300 MPa ± 100 MPa).

The minimum internal bond value for the P2 particleboard is set at 0.35 MPa in the EN 312 standard. According to Table 6, Table 7 and Table 8, it can be seen that this minimum value was clearly exceeded (in the range from 0.40 to 0.49 MPa, i.e., exceeding the values by 14% to 40%), with observable strength increasing with an increase in the content of added investigated wood species in the particleboard core layer. Exceeding the internal bond values is in line with the common trend requirements for current particleboard quality.

In general, following the experiments presented in this paper, it can be stated that the partial use of alternative wood raw materials in the particleboard core layer is a viable option. Testing of the mentioned mechanical properties of the investigated particleboard was carried out on a statistically sufficiently large set, and it demonstrated the suitability of larch, alder, and birch for their use as alternative wood raw materials in the particleboard core layer in proportions of 10, 15, or 20%.

Some researchers conducted research of larch, birch, and alder wood species for wood composite production from slightly different perspectives, but their research studies’ conclusions confirmed more or less the results of our research. Nemlį [60] evaluated the mechanical properties of particleboard laboratory manufacturing from alder (*Alnus glutinosa*, subsp. *Barbata*) wood. He aimed to evaluate the modulus of rupture, modulus of elasticity, and internal bond strength as well as selected physical properties (thickness swelling). His research included a pure 100% particleboard from alder and this is probably a too optimistic consideration of wood composition for particleboard, given the not very large stocks of alder wood in forests in Europe and elsewhere in the world. The results demonstrate that alder particleboard properties exceeded the EN standards’ requirements for the internal bond, modulus of elasticity, and static bending, although the thickness swelling values were poorer than the requirements [60]. This confirms our results that the presence of alder particles does not impair the particleboard quality. Other studies have already worked with alder wood proportions in particleboard, which is a more sober look at the representation of alder in particleboard. There were investigations into the wood particles of gray alder (*Alnus incana* (L.) Moench) in a mixture, and these alder particles were evaluated as acceptable for the purpose of particleboard production [61]. Alder particles were able to withstand even non-wood component mixtures, and these results are in line with our research.

Laskowska and Mamiński [62] mentioned that any new particles should be well bonded together with the UF adhesive in the newly prepared particleboard, and the particleboard mechanical properties could be decreased, e.g., due to the less narrow shape of wood particles and their larger area. We confirm their conclusions, although this was not our case. We used industrially produced standard particles with the correct shape and area; therefore, there was no decrease in the bending strength in our particleboard.

Other authors also confirm that alder, birch, and larch are suitable for particleboard production from various perspectives. Salca [63] pointed out in her research that black alder as a less-utilized wood species has been shown to have considerable potential for industry so that its use can successfully reduce the use of other wood species. Her review offers a contour of the potential of black alder as a wood raw material, and she emphasizes the application and alder wood properties in terms of particleboard production and its process optimization. Razinkov and Ishchenko [64] looked at the issue from the perspective of the shortcoming of particleboard for the production of cabinet furniture mainly caused by the lack of wood raw materials. Their research included a couple of wood species not generally used for particleboard production (mainly birch, alder, pine, aspen, and some others), and alder and birch came out positively from this research.

Pazio and Boruszewski [65] present the results of research on the effect of the addition of particles obtained from European larch wood (*Larix decidua* Mill.) on selected properties of particleboard in comparison to boards of the same structure based on typical industrial raw material uses by the wood-based panels industry. The slight differences were proven in the tests of the internal bond, modulus of rupture, modulus of elasticity in static bending, soaking in water, thickness swelling after 2 and 24 h, and the density profile. However, this was caused by the excessive volume of larch wood used in the entire particleboard volume (50%), so our research using up to 20% larch, even just in the particleboard core layer, is more convincing.

These findings can be considered interesting and useful from the point of view of our research. The decrease in mechanical properties or the reduction in the physical properties of particleboard in some cited works are justified by them and we think, based on our experiments, that this could be due to the higher toughness of some wood particles, e.g., birch, but also larch, even though the shape and slenderness of the particles were identical for all investigated wood species. However, the density of the wood species, their internal structural bonds, and also the adhesiveness and stickiness of such particles play a certain role, and small changes in the properties of particleboard are visible [66].

Analysis of the physical and mechanical properties of particleboard using three lesser-used European wood species in their core layer also verified the correctness of the wood chipping process settings and the correct optimization of the pressing parameters, which do not differ from common industrial parameters and therefore do not incur any increased costs for their modification [67].

### 3.3. Density Profiles of Particleboard

**Figure 3:**
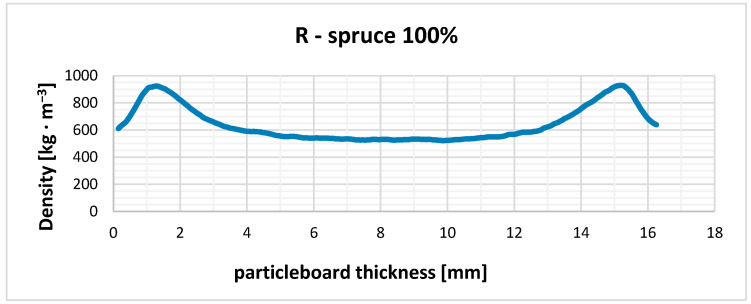
Density profile of particleboard consisting exclusively of spruce particles in all three layers (comparison board—R).

**Figure 4:**
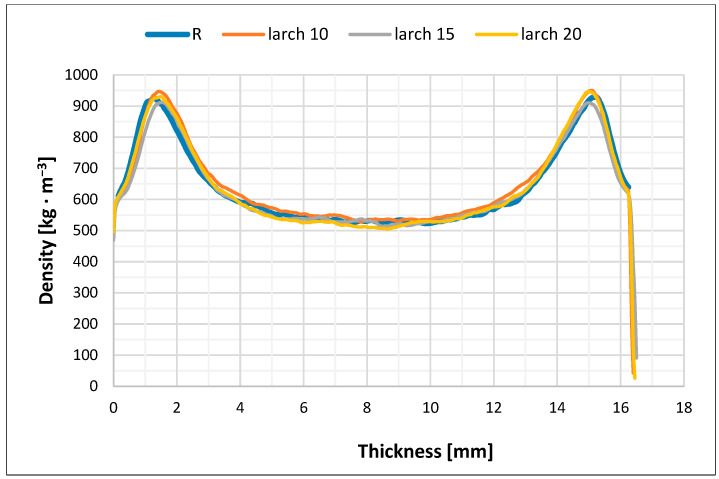
Density profile of particleboard consisting of refined spruce particles in surface layers and coarse particles of larch (10%, 15%, and 20%) and spruce (balance up to 100%) in core layer (L10–L20).

**Figure 5:**
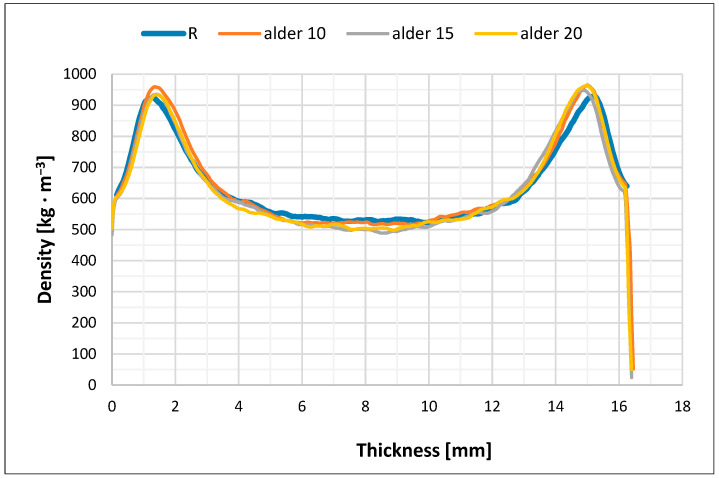
Density profile of particleboard consisting of refined spruce particles in surface layers and coarse particles of alder (10%, 15%, and 20%) and spruce (balance up to 100%) in core layer (A10–A20).

**Figure 6:**
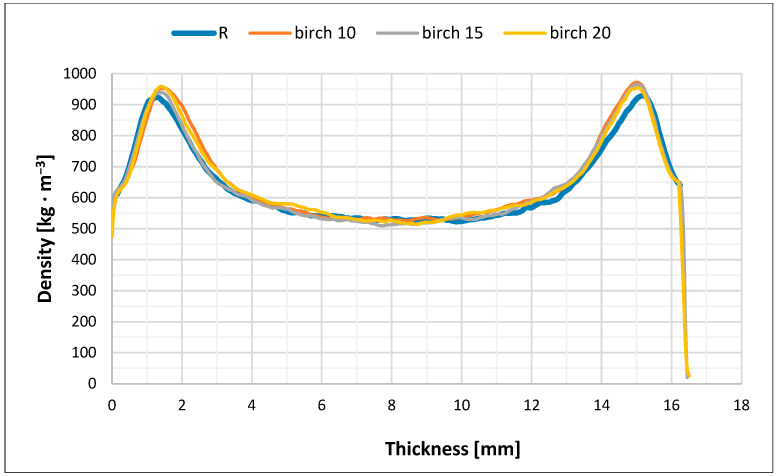
Density profile of particleboard consisting of refined spruce particles in surface layers and coarse particles of birch (10%, 15%, and 20%) and spruce (balance up to 100%) in core layer (B10–B20).

The measured density profiles of the reference pure spruce board (R) and particleboard based on three wood species in their core layer are shown in Figure 3, Figure 4, Figure 5 and Figure 6.

The density profile or the density distribution in the cross-section of the particleboard along the thickness (in layers parallel to the board surface) are a function of several factors, primarily the pressing process technique, the optimization of which can be one of the most effective measures to increase particleboard quality and to use existing reserves in their production. When producing our particleboard, we considered that the density profile of the particleboard significantly affects many of its strength properties, and certain properties, especially bending strength, can be significantly improved by the correct density of the surface layers of particleboard (higher).

The achieved difference in the density of the layers, which was also preserved in the finished particleboard, was caused by the different level of hydrothermal conditions during their densification. We were aware that our pressing had to be identical in all cases, as well as optimal in terms of uniform particleboard quality, because any asymmetry in the achieved density profile of particleboard, either in the distance of the maximum density from the board surface or from the core (neutral axis) or in the value of the maximum density, would mean an asymmetric construction of the finished particleboard, thus creating a potential source of uneven stresses in the particleboard, which could lead to its deformation [46].

Our four types of particleboards were prepared for furniture purposes (type P2), and, therefore, we adapted the pressing method for this type of particleboard. In order to create a density profile with a high density at the surface and a relatively lower density in the core layer of the particleboard, we had to achieve a low resistance to densification of the surface layer during pressing and the highest possible resistance to densification of the core layer. It is clear from Figure 3, Figure 4, Figure 5 and Figure 6 that this was achieved in all cases of pressed particleboard. The gradual heating of the particleboard carpet from the surface to its center with heat from the heated pressing plates of the press at a selected temperature (240 °C) also contributed to fulfilling this requirement. It is obvious that smaller and finer particles in the surface layers, due to faster plasticization, offered less resistance to compaction than the larger core particles.

High temperature pressing can be considered the most important technological operation in the entire technological process of particleboard production with the three tested wood species that we carried out. Our goal was to achieve the required densification of the particleboard layers to a specified thickness, and thus, the density and fixation of the particles in the layers by hardening the adhesive. Our pressing took place under the combined action of heat and pressure, while all the particles (including the core ones with proportions of the three tested wood species) coated with the adhesive mixture were plasticized and densified. This process was certainly accompanied by complex physical and chemical actions that took place in the interaction of heat and pressure with the moisture of the particleboard and its other properties. All factors and components acting in the pressing process contributed to the final particleboard construction and therefore also to their density profiles and particleboard properties. We adjusted the density speed of the particleboard carpets (and therefore the closing speed of the press) so that we achieved particleboard quality P2 and more or less followed the pressing diagram commonly used for the production of particleboard of the type P2 in practice.

From Figure 3, Figure 4, Figure 5 and Figure 6, it is clear that there is probably a connection between our pressing temperature (240 °C) and our rate of particleboard densification because the higher pressing temperature we chose (copying current trends in particleboard pressing temperatures in practice) caused faster heat transfer to the interior of the pressed particleboard and more intensive plasticization of the particles. We can see this in all cases in particleboard at a depth of about 1.1 to 1.2 mm from its surface when the highest densities are achieved, at around 920–950 kg/m^3^, or in the case of larch, from 900 kg/m^3^. A similar situation can be observed from the opposite side of particleboard; the achieved densities are again around 925–955 kg/m^3^, or in the case of larch, from 900 kg/m^3^. The mechanical properties of all three types of particleboard (Table 6, Table 7 and Table 8) showed that their properties were sufficient for particleboard type P2 and, therefore, that the selected pressing diagram and the achieved density profile were acceptable. The minimum density achieved in all three cases examined was around 500 kg/m^3^, with a minor exception in the case of the use of alder particles (amount 15%), and again this was a sufficient density to achieve the correct values of tensile strengths perpendicular to the plane of all particleboard, which exceeded the required value of 0.35 MPa with sufficient margin.

We assume that the pressing temperature we chose (240 °C) could also have led to such a density profile because we ensured appropriate moisture conditions in the particleboard mat. Our pressing temperature did not cause the undesirable effect that the clean spruce surfaces of the particleboard mat would have dried out quickly, as the strength of the adhesive mixtures between the particles would have been compromised, and the necessary densification of both surface layers would not have occurred. This is in accordance with the theoretical ideas of Maloney [1] and also with the more recent observations of Sackey et al. [68], who also state that short-term exposure to higher temperature does not cause the undesirable effect of rapid drying of the particles and the deterioration of the quality of the produced particleboard. Such weaker bonded surface layers would tend to have higher springback, and the particleboard would also have a porous and non-closed surface. It can be seen from Figure 3, Figure 4, Figure 5 and Figure 6 that this did not happen; the strength of the surface layers was sufficient, which was confirmed by the modulus of rupture and modulus of elasticity tests when the required values of 11 MPa and 1600 MPa (for board type P2) were exceeded in all cases. These observations of ours are following Korai [69], who comprehensively analyzed the bending properties of commercial three-layer particleboard to understand the intricate relationships between density profiles and bending properties. In his case as well, obviously, the particleboard exhibited a heightened influence of the outer layer density on the bending properties. The influence of the outer layers on the bending properties varied among his particleboards. The complexity in the mechanism of the bending properties was attributed to intricately intertwined factors: density, long-wood-fiber strength, and the face-to-core ratio. This study [69] highlights the multifaceted nature of bending properties and density profiles, emphasizing the complexities among these factors.

Gamage [70] state in their publication that the vertical density profile influences particleboard properties including flexural strength and its dimensional stability and fastening capacity. Like us, they were aware that it is important to influence the formation of the vertical density profile, altering processing variables, to achieve optimum particleboard properties. Their paper presents an attempt to develop a model to predict the formation of the vertical density profile of hardwood residue particleboard compared to processing parameters, using the theory of experimental design, and the advantages of such a model in optimizing particleboard properties are also discussed.

Harless et al. [71] also correctly stated that certain mechanical properties of particleboard panels depend on the density variations that occur through the panel thickness (density profile). Particleboard density profiles result from the felting and hot-pressing operations. Repeatedly altering a commercial particleboard manufacturing process to produce a predetermined density profile is undesirable from economic and production standpoints. An analytical tool to predict density profile as a function of the manufacturing processes was created by them. Similarly, as in our case, a multilayer description of the density gradients resulting from the felting particleboard process provides input for this research. Inputs for the pressing process include platen temperature and a press closing rate. This result conforms to general expectations, and they confirmed the results we achieved.

In this context, we would like to express the opinion that the research into such a particleboard pressing process and the creation of the correct density profile of particleboard for a specific type (e.g., P2) will retain their permanent relevance at least for the reason that with any change, such as in our case, the partial change in the used wood raw materials or other input raw materials, as a result of the development of new adhesive mixtures, technological processes, and other changes, etc., there will be a need to optimize the pressing process in changed conditions and thus also address the correct density profile of the particleboard.

## 4. Conclusions

- The proportions of 10, 15, and 20% of particles made from three lesser-used European species (alder, larch, and birch) in the particleboard core layer are recommended based on the tests carried out.
- The developed, manufactured, and tested particleboards with such proportions in the core layers are adequate in terms of their achieved quality, and the replacement of part of the traditional spruce particles with these lesser-used European species in the particleboard core layer is possible.
- The proportions of 10, 15, and 20% of particles from alder, larch, and birch in the particleboard core layer do not cause any difficulties in particleboard production.
- Thickness swelling of particleboards after 2 and 24 h is reliably below the conventional limit for all monitored particle ratios and it is reliably satisfactory.
- Water absorption values after 2 and 24 h are completely at the level of particleboard made from pure spruce particles, and therefore they can be reliably used in interiors under the same conditions as conventional particleboard.
- The minimum value of modulus of rupture for the P2 particleboard prescribed by the standard EN 312 is set at 11.0 MPa, and it was met for all proportions of the examined wood species in the particleboard core layer. The addition of birch particles in the core layer shows better bending strength than unmodified spruce particleboard at all three concentrations. This is probably due to the internal structure of birch as a hardwood with a higher density which, in combination with the used adhesive, created a stronger and more compact board material during the board-forming process.
- The minimum internal bond value for the P2 particleboard is set at 0.35 MPa in the EN 312 standard. This value was clearly exceeded (in the range from 0.40 to 0.49 MPa, i.e., exceeding the values by 14% to 40%), with the observable strength increasing with an increase in the content of added investigated wood species in the particleboard core layer.
- The density profile measurements confirmed the correctness of the pressing diagram used for the production of all tested particleboard types. In order to create a density profile with a high density at the surface and a lower density in the core layer of the particleboard, we achieved a low resistance to densification of the surface layer during pressing and the highest possible resistance to the densification of the core layer.
- Particles made from three lesser-used European wood species (alder, birch, and larch) are usable in the amount of 10, 15, and 20% in the particle mixture in the particleboard core layer, and after incorporating them into the particleboard structure by pressing, together with spruce particles, they can be involved in a high-quality particleboard for furniture purposes (P2).

## Figures and Tables

**Table 1:** Properties of urea–formaldehyde (UF) adhesives.

Quality Parameters	Unit	Method	KRONORES CB 4005 D	KRONORES CB 1637 D
Solid content	%	EN 827 [36]	66.01	67.42
Ford cup viscosity, 4 mm/20 °C	s	EN ISO 2431 [37]	77	87
pH value	-	EN 1245 [38]	9.04	8.54
Gel time at 100 °C	s	Kronospan chloride test	80	34

**Table 2:** Individual types of manufactured particleboard.

Variant	Amount of Tested Wood Species in Particleboard Core Layer, *w*/*w* (%)	Board Type
Particleboard core spruce 100%	-	Reference—R
Particleboard core spruce 90%, alder 10%	10	A10
Particleboard core spruce 85%, alder 15%	15	A15
Particleboard core spruce 80%, alder 20%	20	A20
Particleboard core spruce 90%, birch 10%	10	B10
Particleboard core spruce 85%, birch 15%	15	B15
Particleboard core spruce 80%, birch 20%	20	B20
Particleboard core spruce 90%, larch 10%	10	L10
Particleboard core spruce 85%, larch 15%	15	L15
Particleboard core spruce 80%, larch 20%	20	L20

**Table 3:** Physical properties of the control particleboard (R) and of the particleboard containing particles from a mixture of spruce and tested wood species particles made from alder—A10–A20.

Property of Particleboard		Particleboard Core			
		Spruce 100%	Spruce 90% Adler 10%	Spruce 85% Adler 15%	Spruce 80% Adler 20%
Density	(kg/m^3^)	625 (15) ^1^	626 (16)	627 (11)	628 (13)
Thickness swelling after 2 h	(%)	3.89 (3)	4.23 (2)	3.61 (1)	3.42 (1)
Thickness swelling after 24 h	(%)	18.34 (4)	19.50 (5)	18.64 (3)	19.20 (3)
Water absorption after 2 h	(%)	19.35 (3)	17.33 (5)	18.95 (2)	18.56 (2)
Water absorption after 2 h	(%)	61.75 (6)	59.42 (9)	63.14 (8)	63.98 (8)

^1^ Standard deviations are in parentheses.

**Table 4:** Physical properties of the control particleboard (R) and of the particleboard containing particles from a mixture of spruce and tested wood species particles made from birch—B10–B20.

Property of Particleboard		Particleboard Core			
		Spruce 100%	Spruce 90% Birch 10%	Spruce 85% Birch 15%	Spruce 80% Birch 20%
Density	(kg/m^3^)	625 (15) ^1^	632 (16)	624 (20)	625 (19)
Thickness swelling after 2 h	(%)	3.89 (3)	3.43 (1)	2.83 (1)	3.28 (1)
Thickness swelling after 24 h	(%)	18.34 (4)	21.70 (2)	16.07 (3)	13.46 (2)
Water absorption after 2 h	(%)	19.35 (3)	21.14 (3)	17.15 (2)	15.82 (4)
Water absorption after 2 h	(%)	61.75 (6)	67.70 (5)	56.23 (6)	58.22 (7)

^1^ Standard deviations are in parentheses.

**Table 5:** Physical properties of the control particleboard (R) and of the particleboard containing particles from a mixture of spruce and tested wood species particles made from larch—L10–L20.

Property of Particleboard		Particleboard Core			
		Spruce 100%	Spruce 90% Larch 10%	Spruce 85% Larch 15%	Spruce 80% Larch 20%
Density	(kg/m^3^)	625 (15) ^1^	624 (13)	626 (9)	627 (8)
Thickness swelling after 2 h	(%)	3.89 (3)	4.10 (1)	3.88 (2)	4.81 (1)
Thickness swelling after 24 h	(%)	18.34 (4)	19.16 (2)	22.02 (3)	21.25 (2)
Water absorption after 2 h	(%)	19.35 (3)	17.75 (2)	22.27 (5)	20.33 (3)
Water absorption after 2 h	(%)	61.75 (6)	57.43 (7)	60.08 (7)	66.55 (6)

^1^ Standard deviations are in parentheses.

**Table 6:** Mechanical properties of the control particleboard (R) and of the particleboard containing particles from a mixture of spruce and tested wood species particles made from alder—A10–A20.

Property of Particleboard		Particleboard Core			
		Spruce 100%	Spruce 90% Adler 10%	Spruce 85% Adler 15%	Spruce 80% Adler 20%
Modulus of rupture	(MPa)	11.2 (1) ^1^	12.2 (1)	11.8 (1)	11.6 (1)
Modulus of elasticity	(MPa)	2255 (61)	2281 (75)	2241 (154)	2238 (126)
Internal bond	(MPa)	0.44 (0.03)	0.40 (0.02)	0.41 (0.04)	0.43 (0.03)

^1^ Standard deviations are in parentheses.

**Table 7:** Mechanical properties of the control particleboard (R) and of the particleboard containing particles from a mixture of spruce and tested wood species particles made from birch—B10–B20.

Property of Particleboard		Particleboard Core			
		Spruce 100%	Spruce 90% Birch 10%	Spruce 85% Birch 15%	Spruce 80% Birch 20%
Modulus of rupture	(MPa)	11.2 (1) ^1^	12.4 (1)	12.5 (1)	12.9 (2)
Modulus of elasticity	(MPa)	2255 (61)	2309 (114)	2294 (189)	2378 (80)
Internal bond	(MPa)	0.44 (0.03)	0.44 (0.04)	0.46 (0.02)	0.48 (0.01)

^1^ Standard deviations are in parentheses.

**Table 8:** Mechanical properties of the control particleboard (R) and of the particleboard containing particles from a mixture of spruce and tested wood species particles made from larch—L10–L20.

Property of Particleboard		Particleboard Core			
		Spruce 100%	Spruce 90% Larch 10%	Spruce 85% Larch 15%	Spruce 80% Larch 20%
Modulus of rupture	(MPa)	11.2 (1) ^1^	11.4 (1)	11.3 (1)	11.4 (1)
Modulus of elasticity	(MPa)	2255 (61)	2259 (73)	2266 (155)	2254 (127)
Internal bond	(MPa)	0.44 (0.03)	0.49 (0.03)	0.43 (0.04)	0.49 (0.02)

^1^ Standard deviations are in parentheses.

## Data Availability

The original contributions presented in this study are included in the article. Further inquiries can be directed to the corresponding author(s).
